# PDMSA: A Web‐Based Tool for Pan‐Cancer Survival Analysis Using DNA Methylation Levels as Biomarkers

**DOI:** 10.1002/ggn2.202500069

**Published:** 2026-03-14

**Authors:** Weiwei Guo, Ying Shi, Shanshan Wu, Hong Yang, Anqi Lin, Peng Luo

**Affiliations:** ^1^ Department of Oncology Zhujiang Hospital The First School of Clinical Medicine Southern Medical University Guangzhou China; ^2^ Department of Oncology Zhujiang Hospital Southern Medical University Guangzhou China; ^3^ Department of Microbiology State Key Laboratory of Emerging Infectious Diseases Carol Yu Centre For Infection School of Clinical Medicine Li Ka Shing Faculty of Medicine The University of Hong Kong Hong Kong China

**Keywords:** biomarker, cancer, DNA methylation, R Shiny, survival analysis

## Abstract

DNA methylation levels are intimately associated with tumor development, progression, and therapeutic outcomes. Accurate analysis of the relationship between DNA methylation levels and tumor prognosis facilitates comprehensive investigation of tumor development mechanisms, enabling optimization of clinical decision‐making and subsequent enhancement of cancer patient survival rates. However, current web‐based tools for analyzing tumor methylation levels and survival prognosis exhibit significant limitations. We have developed a web‐based tool called Pan‐cancer DNA Methylation Survival Analysis (PDMSA) implemented in Shiny, which integrates DNA methylation data and clinical information from large public databases (TCGA and GEO). PDMSA currently encompasses tumor DNA methylation data from 30 TCGA datasets and 15 GEO datasets, consisting of 16 205 211 records that span 39 cancer types, 45 datasets, 19 909 genes, and 8369 samples. The tool executes prognostic Kaplan–Meier survival analysis and Cox regression analysis utilizing two distinct cutoff value grouping methods, offering customizable visualization options for the results. As a user‐friendly analytical platform, PDMSA serves as a comprehensive tool for biomedical researchers to investigate the relationship between methylation levels at specific gene loci and tumor survival outcomes, thereby facilitating the advancement of precision medicine in oncology. Access PDMSA at robinl‐lab.com/PDMSA.

## Graphical abstract

PDMSA is a web‐based tool for exploring DNA methylation biomarkers across 39 cancer types. It integrates data from 8369 samples and performs survival analysis via Kaplan–Meier and Cox regression. With PDMSA, users can assess the impact of methylation on tumor survival outcomes, aiding the investigation of tumor development mechanisms. Access the tool at: robinl‐lab.com/PDMSA.

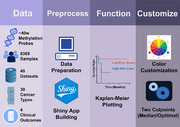

**Abbreviations**
CI: confidence intervals
GEO: gene expression omnibus
HR: hazard ratios
PDMSA: pan‐cancer DNA methylation survival analysis
TCGA: the cancer genome atlas

## 1 Introduction

Tumors, characterized by the infinite proliferation of cells out of control [1], have undoubtedly become one of the most important causes of damage to human health. In 2021, cancer accounted for 17% of all deaths and remains the second leading cause of death after heart disease. However, it is the leading cause of death among women aged 40–79 and men aged 60–79 [2]. The emergence of tumor predictive molecular markers represents a significant milestone in oncology, enabling more efficient tumor diagnosis and treatment, thereby allowing clinicians to significantly improve patient survival outcomes. Effective tumor predictive molecular markers not only facilitate early tumor screening but also guide treatment planning for diagnosed patients and provide accurate prognostic information [3, 4]. Therefore, the identification of reliable tumor predictive markers remains an urgent clinical need. DNA methylation levels, as emerging tumor biomarkers, demonstrate tremendous potential for biomedical researchers in studying tumor prognosis [3, 4, 5, 6].

DNA methylation is a core epigenetic mechanism that modulates gene expression by modifying gene regulatory elements without altering the DNA sequence [7]. Since the discovery of widespread DNA methylation abnormalities in cancer in the 1980s [8], a broad consensus has emerged that aberrant methylation represents a key molecular hallmark of cancer, implicated throughout the entire process of tumor initiation, progression, metastasis, and therapy resistance [9]. Specifically, hypermethylation of CpG islands in promoter regions is a major mechanism for silencing tumor suppressor genes, while global hypomethylation can lead to genomic instability and activation of proto‐oncogenes. Together, these two patterns drive malignant transformation [8].

As a highly promising class of biomarkers, DNA methylation patterns demonstrate unique advantages in molecular subtyping, prognostic assessment, and therapeutic response prediction for tumors [10]. Its strengths lie in the stability of methylation signals, potential for early detection, and frequent direct association with specific pathological mechanisms. For instance, in high‐grade serous ovarian carcinoma (HGSC) cells, a single unmethylated RAD51C allele is sufficient to restore homologous recombination repair (HRR) and confer PARP inhibitor (PARPi) resistance in patient‐derived xenograft (PDX) models; conversely, heterogeneous meRAD51C in PDX models leads to gene silencing and homologous recombination deficiency (HRD), thereby sensitizing these models to PARPi treatment [11]. This case exemplifies how methylation biomarkers can mechanistically explain and predict therapeutic responses [9].

Given its fundamental role in tumor biology and the growing body of evidence supporting its clinical translation, DNA methylation levels at specific loci are widely regarded as a robust and emerging molecular marker for assessing tumor aggressiveness, recurrence risk, and patient survival rates.

However, the utilization of DNA methylation levels for prognostic analysis of cancer patients still faces numerous challenges. Although large public databases such as the Gene Expression Omnibus (GEO) [12] and The Cancer Genome Atlas (TCGA) [13] are available for biomedical researchers to extract and utilize, standardization methods across different laboratories and technological platforms vary considerably, leading to limited data comparability; Additionally, bioinformatics analysis requires researchers to possess a certain level of programming language proficiency [14], which limits in‐depth exploration by some researchers. Even for researchers proficient in programming languages, each methylation sample contains thousands to tens of thousands of CpG sites [12], resulting in large data volumes and time‐consuming analyses; From a methodological perspective, there are numerous methylation data preprocessing algorithms and prognostic analysis algorithms with inconsistent calculation standards [15], making the selection of appropriate analytical methods a challenge for prognostic analysis.

Fortunately, numerous excellent databases and online methylation analysis tools are now available to assist medical researchers in data integration, analysis, and generating sophisticated visualization results [16]. For instance, MethHC [17] facilitates exploration and visualization of the correlation between DNA methylation and microRNA expression. Wanderer [18] allows users to query specific genes, view their methylation levels, and provides data display functions that enable comparison of lineages between tumor and normal samples. GENT2 [19] incorporates gene expression data from 72 different tissues while simultaneously providing prognostic information and survival analysis for relevant genes. These well‐designed tools have significantly advanced research on the relationship between DNA methylation levels and tumors.

Unfortunately, as of now, existing methylation analysis tools predominantly focus on studying the relationship between methylation levels and tumor occurrence, while research on tumor methylation levels and survival prognosis remains insufficient, with limited analytical tools and incomplete functionality. For example, MethSurv [20], based on the TCGA database, employs Cox proportional hazards models for survival analysis but offers relatively limited data and analytical methods; MethHC2.0's [21] survival analysis module provides Kaplan–Meier survival analysis, but the available survival analysis methods require further supplementation, and intuitive data visualization remains an area for improvement.

**FIGURE 1:**
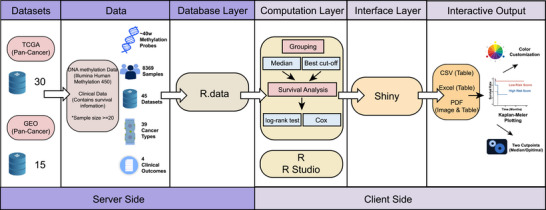
PDMSA workflow. PDMSA (v1.0.0) systematically extracts DNA methylation data and survival information from TCGA and GEO databases, selects datasets containing a minimum of 20 samples, comprising 39 cancer types, 45 datasets, 19 909 genes, and 8369 samples. Users can conduct comprehensive survival analyses via R and RStudio, utilizing either median or optimal cutoff values to perform log‐rank test and univariate Cox regression analysis, with an interactive interface implemented through Shiny, PDMSA generates publication‐quality Kaplan–Meier curves with comprehensive customization options. Abbreviations: GEO: Gene Expression Omnibus; TCGA: The Cancer Genome Atlas; PDMSA: Pan‐cancer DNA Methylation Survival Analysis.

As a result, our team has developed a web‐based tool, pan‐cancer DNA methylation survival analysis (PDMSA), which utilizes DNA methylation levels as tumor biomarkers and focuses on pan‐cancer prognostic analysis. This tool aims to optimize performance in analyzing tumor methylation levels and survival outcomes by incorporating a broader range of data. It places a stronger emphasis on the relationship between tumor methylation levels and survival prognosis, offering a variety of analytical options and a more user‐friendly design. We anticipate that this tool will help maximize the value of public databases, assisting clinicians and researchers in further investigating the mechanisms of tumor development and providing candidate methylation biomarkers for clinical translational research (Figure 1)

## 2 Methods

### 2.1 Data Collection and Preprocessing

Comprehensive DNA methylation profiles of human tumors were systematically obtained from public databases, including TCGA [13] and GEO [12]. All datasets were selected based on the following criteria: (1) data were generated using methylation microarray platforms (Illumina 450K or EPIC); (2) the minimum tumor sample size was 20; (3) complete survival information was available for at least 20 patients. The collected DNA methylation data were subjected to appropriate normalization procedures. Our team downloaded 15 datasets containing DNA methylation and clinical data from the GEO database using the GEOquery package [22] and acquired 30 datasets from the TCGA database using the TCGAbiolinks package [23]. All DNA methylation data were normalized using the “champ.norm” function in the ChAMP package [15]. Prior to downstream analysis, methylation profiles with missing values were filtered out to maintain data integrity and ensure analytical reliability.

Large‐scale, multi‐center cohorts are widely regarded as the gold standard for constructing robust prognostic models with clinical translational potential, particularly for epigenome‐wide association studies (EWAS), which are more susceptible to environmental influences [24, 25]. However, acquiring large sample sizes in the initial stages of research often poses practical challenges, especially in studies focused on specific tumor subtypes, exploratory biomarkers, or investigations with limited resources. It is within this context that the present study was designed as a rigorously controlled, hypothesis‐generating preliminary exploration. Our goal with PDMSA is to screen for a broader range of potential targets, expand the scope of investigation for researchers, and ensure that no promising candidate is prematurely excluded.

Our selection of a minimum sample size of 20 cases was determined through statistical validation and well‐established precedents in the literature. First, during the study design phase, we conducted a sample size estimation based on survival analysis [26] is with a significance level of *α* = 0.05 (two‐sided) and 80% statistical power, at least 15–18 samples per group are required to detect a prognostic effect with a high hazard ratio (HR ≥ 2.5). Our target of 20 cases per group slightly exceeds this theoretical threshold, providing an additional buffer to enhance model stability. Second, when constructing a limited multivariable model, we strictly adhered to classical recommendations in prognostic research methodology [27], ensuring that each parameter estimated corresponds to an adequate number of outcome events. In our preliminary model, we limited the number of variables to 2–3 core predictors, thereby maintaining a ratio of events (e.g., death or recurrence) to variables well above the empirical lower limit of 10:1, which minimizes the risk of overfitting. Third, our sample size aligns with that of widely recognized exploratory studies published in peer‐reviewed journals. For example, in technical validation or subtype‐specific research [28], using approximately 20 samples for initial methylation‐prognosis association analysis is an accepted and feasible practice in the field. Its primary aim is to identify promising candidate biomarkers for subsequent large‐scale validation.

The collected DNA methylation data underwent appropriate normalization procedures. Our team downloaded 15 datasets containing DNA methylation and clinical data from the GEO database using the GEOquery [22] package and acquired 30 datasets from the TCGA database using the TCGAbiolinks package [23]. All acquired DNA methylation data were normalized using the `champ.norm` function in the ChAMP package [15]. Prior to downstream analysis, DNA methylation profiles with missing values were filtered out to ensure data integrity and analytical reliability.

### 2.2 Survival Analysis and Visualization

Methylation data were categorized into two groups using either the median or the optimal cutoff value as the threshold. To ensure the reliability of the analysis, we excluded samples with methylation levels that were too low (below the fifth percentile of all samples) or too high (above the 95th percentile [29]), in order to avoid statistical bias due to insufficient sample sizes. The calculation of the optimal cutoff value was performed using the surv_cutpoint function from the R package survminer (version 0.4.9), with the condition that each subgroup contained at least 5% of the total sample size to ensure statistical stability.

Next, we employed Kaplan–Meier survival curves and used the log‐rank test to assess the significance of group differences. Univariate and multivariate Cox proportional hazards regression models were then applied to analyze the prognostic value of methylation levels, with the results visualized using forest plots. All analyses were conducted using the survival package in R (v3.5.7), where the survfit function was used for Kaplan–Meier analysis and the coxph function for Cox proportional hazards regression. Overall, for each methylation site, we computed the corresponding p‐value of the survival curve, the hazard ratio (HR), and its 95% confidence interval (95% CI) to comprehensively evaluate the association between methylation levels and patient prognosis.

To adjust for the influence of potential clinical confounders, PDMSA integrates multivariate Cox regression analysis. For each dataset, the system extracts core clinical variables from the original clinical annotations, including sex, age, race, ethnicity, and pathological stage. On the “Cox FOREST!” tab of the PDMSA interactive interface, users can select 2–5 covariates to include in the model via the “CUSTOMIZE CLINICAL FACTORS” panel. All categorical variables are encoded as factors before analysis, with fixed reference groups set as follows: sex with “male” as reference, age group with “21–30 years” as reference, race with “White” as reference, ethnicity with “Non‐Hispanic/Latino” as reference, and pathological stage with “Stage I” as reference. Missing values in clinical variables are uniformly labeled as “Unknown” during preprocessing and included as an independent group in model fitting. Samples with missing survival time or status are excluded. All multivariate analyses are performed based on subsets of samples with complete methylation data, survival data, and the selected clinical variables.

To address the issue of multiple testing in high‐dimensional methylation analyses, we have integrated a False Discovery Rate (FDR) correction module based on the Benjamini–Hochberg (BH) method [30]. This module accepts raw p‐values from survival analyses, sorts them in ascending order, and computes adjusted q‐values according to the formula:$q\;\left(i\right)=p\left(i\right)\times \frac{m}{i}$, where m is the total number of tests, and i is the rank of the p‐value. Monotonicity is enforced to ensure that the adjusted values remain non‐decreasing. The results are presented with annotations indicating significance at thresholds of *α* = 0.05 and α = 0.01, helping researchers distinguish robust signals from false discoveries. This feature enhances the statistical rigor of the platform and supports reproducible research in epigenomic biomarker discovery.

### 2.3 Application Implementation

PDMSA is an interactive web‐based analytical platform implemented in R (4.3.1), leveraging the Shiny package (1.7.5) for the development of the user interface and server‐side logic. The core survival analysis functionality is implemented through packages including DT (0.33) and survival (3.5.7). After data normalization, all processed data are stored as Rdata files on the server. A comprehensive list of R packages and their dependencies is provided in Table S1. Figure 1 presents a systematic overview of PDMSA, delineating the workflow from data input to result output, thereby providing users with a comprehensive operational guide.

### 2.4 Statistical Analysis

All statistical analyses in PDMSA were conducted using R software (version 4.3.1). In terms of data preprocessing, DNA methylation data obtained from TCGA and GEO were normalized with the champ.norm function (ChAMP package, version 2.24.0) to minimize technical batch effects, and probes with missing values were removed to maintain data integrity. To reduce statistical bias from extreme values, samples with methylation levels falling below the fifth percentile or exceeding the 95th percentile were excluded from survival analysis. Regarding data presentation, survival outcomes were visualized using Kaplan–Meier curves accompanied by hazard ratios (HR) and 95% confidence intervals (95% CI), while categorical variables were summarized as frequencies and proportions. With respect to sample size, the current iteration of PDMSA comprises 16 205 211 methylation records from 8,369 samples across 45 datasets and 39 cancer types. For each analysis, the effective sample size (n) was defined as the number of patients with complete methylation and survival data, and a minimum of 20 tumor samples was required to achieve 80% statistical power at a two‐sided α level of 0.05 for detecting a HR ≥ 2.5. Survival analysis was performed by generating Kaplan–Meier curves using the survfit function, with inter‐group differences evaluated via the log‐rank test. Both univariate and multivariate Cox proportional hazards regression were carried out using the coxph function; the multivariate models were adjusted for sex, age, race, ethnicity, and pathological stage. Missing clinical data were coded as “Unknown” and treated as a separate category. Methylation levels were dichotomized based on two distinct strategies: the median value and an optimal cutoff identified by the surv_cutpoint function that maximizes survival curve separation, with the constraint that each resulting subgroup contained at least 5% of the total samples. To address multiple comparisons, the Benjamini‐Hochberg procedure was employed to control the false discovery rate, and statistical significance was denoted at adjusted q‐value thresholds of 0.05 and 0.01. The proportional hazards assumption was assessed for key findings using the R survival package. All statistical tests were two‐sided, and *p* values below 0.05 were interpreted as statistically significant. A complete list of R packages and their dependencies is available in Table S1.

## 3 Results

PDMSA incorporates tumor methylation data from 30 datasets obtained from TCGA [13] and 15 datasets from the GEO [12] database, totaling 16 205 211 records, which encompass 39 cancer types, 45 datasets, 19 909 genes, and 8369 samples (Figure 1). All datasets can be queried for detailed information through an interactive table located in the DATA Tab, including cancer type, dataset platform, survival information, and links to corresponding datasets. While addressing diverse analytical needs, PDMSA also enables users to navigate to dataset websites of interest to view additional detailed information for further analysis.

### 3.1 Gene Overview

To enhance users' ability to intuitively compare differences in genomic sites across various cancer types, we invite users to visit the “Gene Overview” module. This module integrates DNA methylation data from both TCGA and GEO databases. Utilizing a bioinformatics analysis pipeline and interactive visualization techniques, it generates cross‐cancer methylation site distribution lollipop charts for each gene. Users first filter genes by risk type (high‐risk, HR > 1, or low‐risk, HR < 1), after which the system dynamically updates the gene list and displays the total number of available probes for each gene based on pre‐computed multi‐cancer survival analysis results. Upon selecting a target gene, PDMSA performs genomic coordinate annotation using the Ensembl database, generating a visualization anchored to the transcription start site (represented by a red block) and the gene body (represented by a black block). In the chart, each lollipop represents a CpG probe. The x‐axis indicates its precise genomic location, while the height of the lollipop on the y‐axis visually represents the number of cancer types in which that site shows a significant association with survival (*p* < 0.05). Through interactive parameter settings, users can customize the display to show the top N most significant probes. The system automatically adjusts the chart layout (single or multiple chromosomes displayed separately) based on the number of chromosome distributions. A dynamic information panel simultaneously displays basic gene information, risk classification, total probe count, covered cancer types, data source, and statistical significance range. This module not only provides rich visual expression but also offers advanced functionalities such as cross‐cancer comparison, hotspot region identification, and genomic context analysis. It supports PDF download, serving as an efficient visualization and analysis platform for researchers to explore cancer‐specific and common patterns in DNA methylation.

### 3.2 Survival Analysis

The core component of PDMSA is its survival analysis function. Users can select built‑in preset genes and their corresponding methylation probes for survival analysis in the “About” tab. The software supports switching between different methylation level groupings and allows customization of Kaplan–Meier survival curve colors. All survival analysis result tables can be saved or printed in various formats (e.g., CSV, Excel, PDF), and result plots can be exported as PDF. In the survival analysis result table, users can view ID, Probe, Gene, Cancer, Dataset, Platform, Survival, CHR, Strand, Feature, CGI, and relevant KM and Cox survival analysis metrics. The complete table is included in Files S1 and S2. In the Cox analysis forest plot table, users can see Term Label, HR (95% CI), and *p*‐value. The complete table is provided in File S3.

We emphasize that the data‐driven optimal cut‐off selection employed by this tool is intended for exploratory analysis. Since the same dataset is used for both cut‐point searching and significance testing, there is an increased risk of Type I error inflation. Therefore, the resulting “optimal” cut‐off and its associated survival difference (*p*‐value) must be considered preliminary findings and require prospective validation in independent cohorts.

### 3.3 Tool Information and User Support

Users can access detailed tutorial videos and solutions to common issues encountered during usage through the ABOUT Tab. Additionally, PDMSA enables users to provide feedback, evaluations, or suggestions to the development team via email or a comment system. The development team commits to carefully considering each piece of feedback, continuously optimizing and maintaining PDMSA, and providing real‐time version updates on the website to ensure ongoing improvement and transparency of the tool.

### 3.4 Case Analysis

**FIGURE 2:**
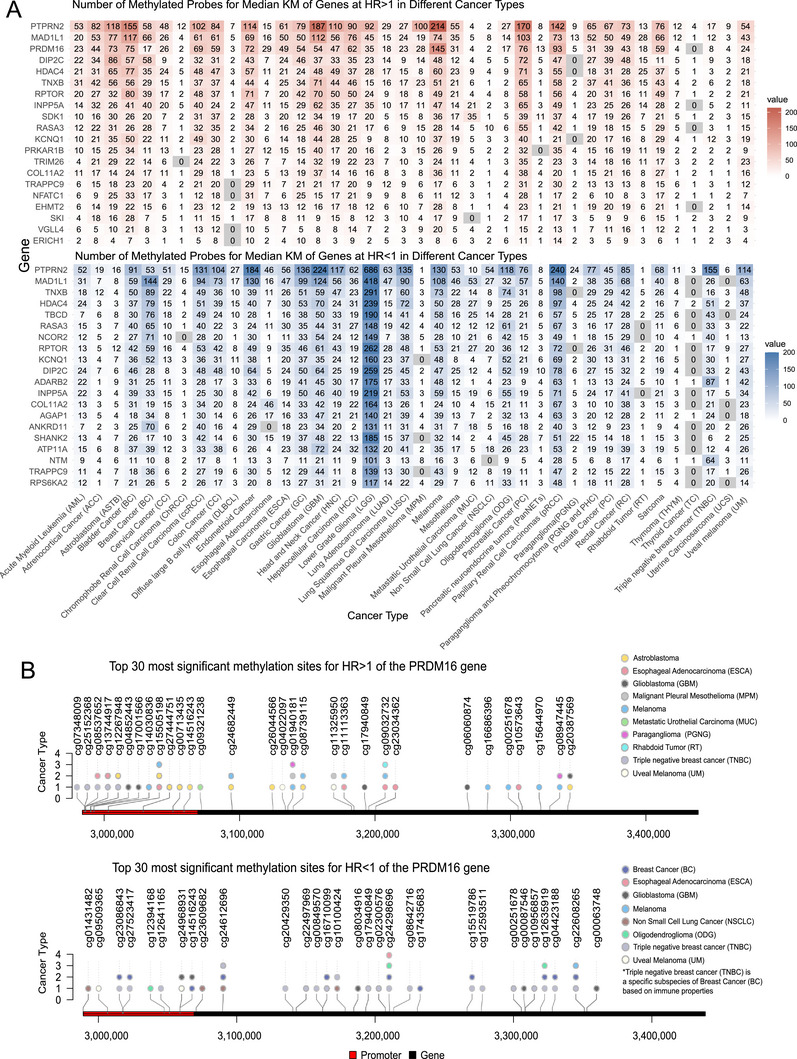
PDMSA Survival Analysis Case Findings. (A) Distribution of Methylation‐Associated Probes Identified through Median–Stratified Kaplan–Meier Analysis across Cancer Types. Through median‐stratified Kaplan–Meier survival analysis, we identified statistically significant probes (*p *< 0.05) and ranked the top 30 genes based on both probe abundance and their prevalence across multiple cancer types. (B) Characterization of the 30 Most Significant Methylation Sites in PRDM16. PRDM16, identified as a methylation‐regulated gene with significant prognostic implications across multiple cancers, exhibits 30 key methylation sites that demonstrate either tumor‐promoting or protective effects, with distinct distribution patterns across various cancer types. Methylation sites correlating with adverse prognosis were primarily clustered within the promoter region, whereas the remaining sites exhibited a dispersed distribution pattern.

Through comprehensive statistical analysis of methylation probes with significant log‐rank test results (*p* < 0.05) in the PDMSA database, and stratification based on the median, we systematically identified the top 30 genes characterized by the highest probe frequency and the widest range of cancer types (Figure 2a). After excluding genes that appeared in both high‐risk and low‐risk probe rankings due to their long gene length, PRDM16 was ultimately selected as a candidate methylation‐regulated prognostic biomarker for experimental validation, as it is associated with poor clinical outcomes in multiple cancer types.

**FIGURE 3:**
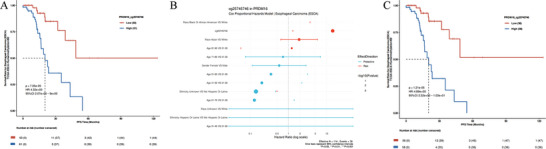
Kaplan–Meier plot of the results of methylation probe cg25745746 of PRDM16 analyzed in PDMSA. (A) Methylation probe for PRDM16, cg25745746, was analyzed by median grouped log‐rank test survival and showed a higher survival rate for hypomethylation in Esophageal Carcinoma (ESCA) (TCGA‐ESCA), in agreement with the study of Peng et al. (B) Multivariate Cox analysis of methylation probe for PRDM16, cg25745746, revealed that non‐Hispanic/Latino patients had a significantly higher risk of death (HR = 0.141, 95% CI: 0.04‐0.45, *p* = 0.001), in agreement with epidemiological data on esophageal cancer (EC) in the U.S. from 1975 to 2018. (C) Methylation probe for PRDM16, cg25745746, was analyzed by optimal cutoff‐based grouped log‐rank test survival, suggesting that the optimal cutoff‐based grouping more accurately identifies patient groups with better prognoses.

We then investigated the 30 differentially methylated sites in the PRDM16 gene that were linked to either poor or good patient outcomes, along with the corresponding cancer types in the PDMSA database (Figure 2b). Methylation sites associated with poor tumor prognosis were primarily located in the promoter region, while other sites exhibited a more scattered distribution pattern, indicating that PRDM16 promoter methylation plays a key role in tumorigenesis and progression, and may serve as a prognostic biomarker, consistent with previous studies. Specifically, methylation analysis of the promoter region revealed significant differences in the PRDM16 methylation pattern between esophageal cancer tissues and normal controls, showing high diagnostic value and suggesting its potential application as a diagnostic biomarker for esophageal cancer [31]. These findings align with the analysis of the PDMSA dataset. Figure 3 displays the methylation analysis of the PRDM16 gene probe, cg25745746, in esophageal cancer extracted from the PDMSA database.

In the ESCA cohort, we first performed univariate Kaplan–Meier (KM) survival analysis to evaluate the methylation level of PRDM16. The results showed that patients in the PRDM16 low‑methylation group had significantly better progression‑free survival than those in the high‑methylation group when the median was used as the cut‑off (*p* = 7.35 × 10^−^^5^), suggesting a potential association between methylation level and prognosis (Figure 3a). However, KM analysis only reflects between‑group differences and does not control for confounders such as age and tumor stage. Therefore, we further employed a Cox proportional hazards regression model to quantify the hazard ratio (HR). The univariate analysis yielded an HR of 4.65 (95% confidence interval: 2.035–10.611, *p* = 2.66 × 10^−^^4^), indicating that the high‑methylation group had a significantly higher risk of death than the low‑methylation group. We hypothesize that hypermethylation of PRDM16 may be an independent risk factor for esophageal cancer and that the risk becomes more pronounced with higher methylation levels. In the multivariate Cox model, we included ethnicity (with three categories: Hispanic/Latino, Not Hispanic or Latino, and Ethnicity Unknown), using Not Hispanic or Latino as the reference group. The results showed that, compared with the reference group, the Ethnicity Unknown group had a significantly lower risk of death (HR = 0.141, 95% CI: 0.04–0.45, *p* = 0.001), whereas the Hispanic/Latino group showed no significant difference in risk (*p* = 0.997) (Figure 3b). Given that our primary finding centers on the association between PRDM16 hypermethylation and poor prognosis, we further speculate that hypermethylation may represent a common risk factor across ethnic groups. Future studies with larger, well‑characterized, and ethnically balanced cohorts are needed to validate the prognostic value of PRDM16 methylation in different ethnic subgroups and to investigate whether its upstream regulatory mechanisms are influenced by ethnicity‑related genetic or environmental factors.

To further explore the biological threshold effect of methylation levels, we compared the analysis results between median‐based grouping and optimal cutoff‐based grouping. The results showed that, both in Kaplan–Meier (KM) analysis and Cox regression analysis (Figure 3c), the optimal cutoff‐based grouping yielded more significant p‐values (KM: *p* = 1.21 × $10^{-5}$ vs. 7.35 × $10^{-5}$; Cox: *p* = 7.16 × $10^{-5}$ vs. 2.66 × $10^{-4}$), suggesting that the optimal cutoff‐based grouping more accurately identifies patient groups with better prognoses.

In investigating the relationship between methylation levels and prognosis, we recommend the following analysis strategy: Given the convenience and speed of median‐based grouping, we first perform Kaplan–Meier survival analysis combined with median‐based grouping for preliminary screening. Subsequently, Cox proportional hazards regression is used for multivariate analysis to control for the effects of confounding factors such as age and ethnicity. For a deeper exploration of specific biomarkers, we recommend using optimal cutoff‐based grouping, determined through statistical testing, as it more accurately reflects the impact of methylation levels on survival prognosis.

## 4 Discussion

PDMSA is a powerful interactive web‐based tool that offers accessibility and operational simplicity, enabling biomedical researchers to rapidly and conveniently investigate tumor development mechanisms in depth, thereby enhancing clinical decision‐making. PDMSA integrates pan‐cancer methylation data and clinical survival information from TCGA [13] and GEO [12] databases, allowing researchers to select datasets and analytical methods according to specific requirements and visualize their results. By offering large‐scale datasets, diverse cancer types, and flexible customized analysis options, PDMSA has the potential to serve as a powerful resource in the field of tumor prognostic analysis. We emphasize that its role is that of a pre‐validation screening aid; thus, findings from PDMSA should be viewed as generating candidates for subsequent rigorous validation within established research paradigms.

To more comprehensively evaluate the innovativeness of PDMSA, we systematically compared it with existing specialized DNA methylation survival analysis tools—MethSurv [20] and MethHC 2.0 [21]—as well as widely used comprehensive multi‑omics/TCGA data portals such as UALCAN [32] and LinkedOmics [33] (Table 1). This extended comparison highlights the integrated advantages of PDMSA. In terms of data breadth and representativeness, unlike tools such as MethSurv [20] and Wanderer [18], which only incorporate TCGA data, PDMSA integrates two major public databases—TCGA and GEO—expanding the analyzable methylation sample size to 8,369 samples covering 39 cancer types. It also supports analysis of four clinical endpoints: overall survival (OS), progression‑free survival (PFS), distant metastasis‑free survival (DMFS), and metastasis‑free survival (MFS). Regarding depth and specialization of analytical methods, compared to tools like MethHC 2.0 [21] and Wanderer [18], which only offer Kaplan–Meier analysis, PDMSA provides Cox regression and a dual‑grouping strategy using both median and optimal cut‑points. In contrast to platforms such as UALCAN [32] and LinkedOmics [33]—where survival analysis is not methylation‑specific, lacks optimal cut‑point options, and is mostly expression‑based—PDMSA delivers a complete multivariate Cox regression workflow centered on methylation levels and allows adjustment for multiple clinical factors. With respect to research efficiency and output, PDMSA's intuitive interactive interface avoids the complex input requirements of tools like MethHC 2.0 [21] and supports interactive exploratory analysis, enabling users to quickly screen and review multiple results through a user‑friendly layout. Particularly noteworthy is PDMSA's “Gene Overview” module, which uniquely generates cross‑cancer methylation site distribution lollipop charts. This visualization method allows intuitive comparison of genomic site differences and helps researchers rapidly identify methylation hotspot regions—an insight not available in existing tools. All statistical results and publication‑ready graphics can be exported with one click into various formats (e.g., CSV, PDF), substantially improving efficiency throughout the entire workflow from analysis to publication. In terms of scientific rigor, PDMSA ensures higher transparency and reproducibility. Developed on the open‑source Shiny framework, its complete code is publicly available on GitHub, making the entire analysis pipeline auditable and reproducible, thereby providing a foundational guarantee for the reliability of research findings. By integrating these systematic advantages, PDMSA successfully establishes a dedicated one‑stop platform for DNA methylation biomarker discovery, evaluation, and validation, filling a gap in the existing tool ecosystem.

**TABLE 1:** Side‐by‐side comparison of PDMSA with other tools.

	PDMSA	MethSurv	MethHC 2.0	UALCAN	LinkedOmics
**Data type**	TCGA methylation data profiled using HM450K array which covers 486 428 CpGs, and GEO methylation data profiled using HM450K array	TCGA methylation data profiled using HM450K array which covers 486 428 CpGs	TCGA methylation: 9736 microarray data; GEO methylation: 17454 microarray data; manually collected and up to 3586 curated data	TCGA multi‐omics data: RNA‐seq, DNA promoter methylation (HM450K), CPTAC proteomics, and selected ChIP‐seq data	TCGA multi‐omics data: mutation, CNA, methylation, mRNA/miRNA expression, RPPA, clinical; CPTAC proteomics data: global, phospho‐, and glycoproteomics data
**Dataset** ^a^	45	25	33	33	32
**Cancer type** ^a^	39	25	33	33	32
**Sample** ^a^	8369	7358	27 190	>10 000	11 158
**Survival outcome** ^a^	4	1	1	1	1
**Optimal cutpoint**	YES	YES	NO	NO	NO
**Cox analysis**	YES	NO	NO	NO	YES
**Color xustomization**	YES	NO	NO	NO	NO
**Multivariable analysis**	YES	YES	NO	YES	NO

^a^ The number of datasets, cancer types, samples, and survival outcomes available in the survival analysis.

Aggregated: Datasets have been aggregated when conducting the survival analysis.

PDMSA has a broader richness in datasets, cancer types, and survival states; it also supports optimal cutoff grouping, Cox regression analysis, and allows for user‐defined.

It is important to emphasize that the methylation survival analysis results provided by PDMSA should be interpreted in the context of the clinical background. While the tool supports multivariate Cox proportional hazards regression for the correction of basic clinical factors, cancer prognosis evaluation still requires a comprehensive consideration of treatment plans, molecular mutations, and other key factors. For example, the PRDM16 methylation sites identified by this tool need to be further validated in subsequent studies for their prognostic value independent of treatment modalities. Additionally, studies have reported that patients with MGMT promoter methylation are more sensitive to temozolomide treatment [34], highlighting the need for methylation analysis to be combined with treatment strategies. Therefore, future research could use PDMSA to screen for candidate biomarkers, followed by multi‐center validation studies incorporating a more comprehensive set of clinical variables.

However, PDMSA has several limitations that warrant discussion. First, regarding dataset scope, PDMSA currently only integrates methylation array data from TCGA and GEO databases; experimental data from numerous publications and other large methylation databases such as EWAS Data Hub [35] remain to be incorporated, suggesting that the dataset is not yet sufficiently comprehensive. Additionally, current analyses have not fully adhered to the standard validation frameworks for biomarker research. Further validation of the clinical reproducibility and statistical power of methylation biomarkers is required in the future. For instance, the REMARK guidelines [36], Item 12, explicitly mandate that biomarker studies should include independent validation cohorts to confirm clinical applicability. However, PDMSA is currently based solely on public datasets and lacks support from local or independent cohorts, which may impact the reproducibility of biomarker validation. Furthermore, the MIAME [37] standards emphasize the integrity of microarray data, including experimental design and sample processing details. The absence of critical information in certain public datasets could compromise the reliability of PDMSA analyses. Given the dynamic updating nature of these databases, our study emphasizes the need to establish a systematic mechanism to incorporate new datasets in a timely manner, a challenge that has also been highlighted in recent comprehensive reviews of multi‐omics applications in pharmaceutical research [38, 39]. Second, PDMSA currently only supports retrieval and analysis within preset datasets and genes, and lacks the capability to process user‐defined data. This limitation may impede researchers with unique sample data from fully utilizing the tool, thereby potentially limiting opportunities for novel oncological discoveries. Future development should consider implementing user data upload and analysis capabilities to enhance the tool's applicability and research value. Third, with respect to survival analysis functions, PDMSA currently only supports median grouping and optimal cutoff value grouping methods, without implementing user‐defined grouping functionality, which limits the flexibility and personalization of survival analysis. In terms of statistical analysis functionality, the current version of PDMSA does not include an automated test for the proportional hazards assumption, primarily to maintain the computational efficiency and responsiveness of this interactive exploratory platform. Given the importance of this assumption for model validity, while PDMSA's results can provide valuable preliminary insights for biomarker screening, we recommend that users conduct supplementary validation of key findings using specialized statistical software (e.g., the survival package in R) before proceeding to in‐depth causal inference or clinical translation studies. Future versions will evaluate the feasibility of incorporating this optional feature.

To address the limitations of current datasets, we have formulated a systematic PDMSA maintenance and update strategy. This strategy includes regularly integrating newly added database resources, continuously optimizing existing functions, and improving tool performance based on user feedback. Additionally, based on our team's experience in developing multi‐domain tumor survival analysis network tools [40, 41], we plan to expand the toolkit, with the goal of providing oncology researchers and clinicians with a comprehensive analysis platform. These tools will support molecular‐level exploration throughout the diagnosis‐to‐treatment process, thereby advancing the application of precision medicine in the field of oncology.

In conclusion, PDMSA, as a user‐friendly analytical platform, provides biomedical researchers with a powerful tool to explore the relationship between methylation levels at specific gene loci and tumor survival outcomes. Despite its limitations, through continuous optimization and user‐driven updates, PDMSA has the potential to become an important resource in the field of tumor epigenetics research, providing reliable data support for both research and clinical practice.

## Author Contributions

Writing – original draft, Y.S., W.W.G., S.S.W, and H.Y.; conceptualization, A.Q.L. and P.L.; investigation, Y.S., W.W.G., S.S.W, and H.Y.; writing – review and editing, Y.S., W.W.G., S.S.W, H.Y., A.Q.L., and P.L.; visualization, Y.S., W.W.G., S.S.W, and H.Y. All authors have read and agreed to the published version of the manuscript.

## Funding

The authors have nothing to report.

## Conflicts of Interest

The authors declare no conflicts of interest.

## Supporting information




**Supporting File 1**: ggn270033‐sup‐0001‐File1.csv

**Supporting File 2**: ggn270033‐sup‐0002‐File2.csv

**Supporting File 3**: ggn270033‐sup‐0003‐File3.csv

**Supporting File 4**: ggn270033‐sup‐0004‐TableS1.pdf

## Data Availability

The datasets analysed during the current study are available in the PDMSA_data repository, https://data.mendeley.com/datasets/fndcfbj25m/1 (DOI:10.17632/fndcfbj25m.1). The underlying code for this study is available in github and can be accessed via this link https://github.com/Shoshana02/PDMSA.
