# Supplementary material for: PDMSA: A Web‐Based Tool for Pan‐Cancer Survival Analysis Using DNA Methylation Levels as Biomarkers
Source: Adv Genet (Hoboken). 2026 Mar 14;7(1):e00069. doi: 10.1002/ggn2.202500069 (PMC13093317; doi:10.1002/ggn2.202500069)
Supplement: Supplementary file 4 — Supporting File 4: ggn270033‐sup‐0004‐TableS1.pdf [file GGN2-7-e00069-s002.pdf]

Supplementary Table 1

Data Acquire
GEOquery
TCGAbiolinks

Data Normalization
ChAMP

Survival Anaylsis
survminer
survival
dplyr

Implementation
shiny
shinyWidgets
shinythemes
shinydashboard
shinydashboardPlus
tidyverse
DT
shinycssloaders
shinyjs
shinyFeedback
purrr
stringr
shinyscroll
shinyhelper
spsComps
readxl
readr
reshape
reshape2
tibble
countup
emayili
shinyalert
